# Discovery of Substituted 5-(2-Hydroxybenzoyl)-2-Pyridone Analogues as Inhibitors of the Human Caf1/CNOT7 Ribonuclease

**DOI:** 10.3390/molecules29184351

**Published:** 2024-09-13

**Authors:** Ishwinder Kaur, Gopal P. Jadhav, Peter M. Fischer, Gerlof Sebastiaan Winkler

**Affiliations:** School of Pharmacy and Centre for Biomolecular Sciences, University of Nottingham, Nottingham NG7 2RD, UK; ishwinder.kaur@ntu.ac.uk

**Keywords:** Caf1, CNOT7, CNOT8, mRNA, deadenylase, inhibitor, nuclease, gene regulation

## Abstract

The Caf1/CNOT7 nuclease is a catalytic component of the Ccr4-Not deadenylase complex, which is a key regulator of post-transcriptional gene regulation. In addition to providing catalytic activity, Caf1/CNOT7 and its paralogue Caf1/CNOT8 also contribute a structural function by mediating interactions between the large, non-catalytic subunit CNOT1, which forms the backbone of the Ccr4-Not complex and the second nuclease subunit Ccr4 (CNOT6/CNOT6L). To facilitate investigations into the role of Caf1/CNOT7 in gene regulation, we aimed to discover and develop non-nucleoside inhibitors of the enzyme. Here, we disclose that the tri-substituted 2-pyridone compound 5-(5-bromo-2-hydroxy-benzoyl)-1-(4-chloro-2-methoxy-5-methyl-phenyl)-2-oxo-pyridine-3-carbonitrile is an inhibitor of the Caf1/CNOT7 nuclease. Using a fluorescence-based nuclease assay, the activity of 16 structural analogues was determined, which predominantly explored substituents on the 1-phenyl group. While no compound with higher potency was identified among this set of structural analogues, the lowest potency was observed with the analogue lacking substituents on the 1-phenyl group. This indicates that substituents on the 1-phenyl group contribute significantly to binding. To identify possible binding modes of the inhibitors, molecular docking was carried out. This analysis suggested that the binding modes of the five most potent inhibitors may display similar conformations upon binding active site residues. Possible interactions include π-π interactions with His225, hydrogen bonding with the backbone of Phe43 and Van der Waals interactions with His225, Leu209, Leu112 and Leu115.

## 1. Introduction

The human Caf1/CNOT7 nuclease is a member of the DEDDh subgroup of the RNAse D family of exonucleases [1,2,3,4,5,6,7]. The enzyme removes the 3′ terminal nucleotide in a distributive manner and selectively recognises poly(A) RNA substrates. As is typical for enzymes of the RNAse D family, enzyme activity is dependent on two Mg^2+^ ions that are coordinated by three Asp (D40, D161 and D230) and a Glu (E42) residue in the active site [4,5,6,7,8,9]. Caf1/CNOT7 is a catalytic component of the human Ccr4-Not complex [10,11,12,13], which is involved in the degradation of cytoplasmic mRNA by the shortening and removal of the poly(A) tail [14,15,16,17,18]. This initiates the removal of the 5′ cap structure and the exposure of the 5′ end of mRNA to degradation by the 5′-3′ exoribonuclease Xrn1 [2,3,4,5].

In addition to their catalytic roles, Caf1/CNOT7 and its paralogue Caf1/CNOT8 also have a structural role in the Ccr4-Not complex by mediating the interaction between the large subunit of the complex, CNOT1, and the second nuclease subunit, Ccr4 (CNOT6/CNOT6L) [3,4,5,10,12,19]. The contribution of the two nuclease subunits is not well understood: it is currently not clear whether the nuclease activities of the Caf1 and Ccr4 subunits are collaborative and inhibition of one subunit impacts on the second [13,20,21,22,23,24], or whether the Caf1 and Ccr4 nucleases have specialised roles [25,26].

In higher eukaryotes, Caf1 is encoded by two paralogous genes: CNOT7 and CNOT8. In human and mouse cells, CNOT7 and CNOT8 have overlapping functions and can partially compensate for loss-of-function of the other paralogue [27,28,29,30]. Both paralogues are required for efficient cell proliferation of breast and gastric cancer cells [27,31]. However, the paralogues also have unique roles. In mice, Cnot7 is a repressor of osteoblast function and deletion of Cnot7 improves bone density [32,33]. Moreover, genome-wide association studies using recombinant inbred mice identified the involvement of Cnot7 gene variants in bone biology [34]. The use of recombinant inbred mouse strains also led to the identification of Ccr4-Not components as modulators of cancer metastasis [35]. Specifically, Caf1/Cnot7 promotes metastatic disease in a murine model of breast cancer [36]. In this case, the role of Cnot7 in promoting metastasis is dependent on its catalytic activity suggesting that inhibitors of Caf1/CNOT7 may reduce the incidence of breast cancer metastasis [36].

Inhibitors of Caf1/CNOT7 offer an alternative approach to genetic methods and can provide valuable starting points for possible subsequent drug discovery programmes. Therefore, to facilitate the discovery and identification of drug-like inhibitors of the Caf1/CNOT7 nuclease, we previously developed a quantitative, fluorescence-based assay using purified components [20,37]. This assay was used to develop Caf1/CNOT7 inhibitors based on the presence of a N-hydroxy-imide moiety [38], which was proposed to coordinate two Mg^2+^ ions in the active sites of the FEN-1 and HIV RNase H ribonucleases [39,40,41,42].

We also used the fluorescence-based assay to identify inhibitors of the Caf1/CNOT7 nuclease by screening a library of compounds [37]. Here, we disclose the structure of the most potent ‘hit’ compound of the screen for drug-like inhibitors, a tri-substituted 2-pyridone, and report the results of a preliminary structure–activity relationship analysis. We also report plausible binding interactions between the most potent analogues and the binding pocket of human Caf1/CNOT7 using molecular docking.

## 2. Results and Discussion

**Figure 1:**
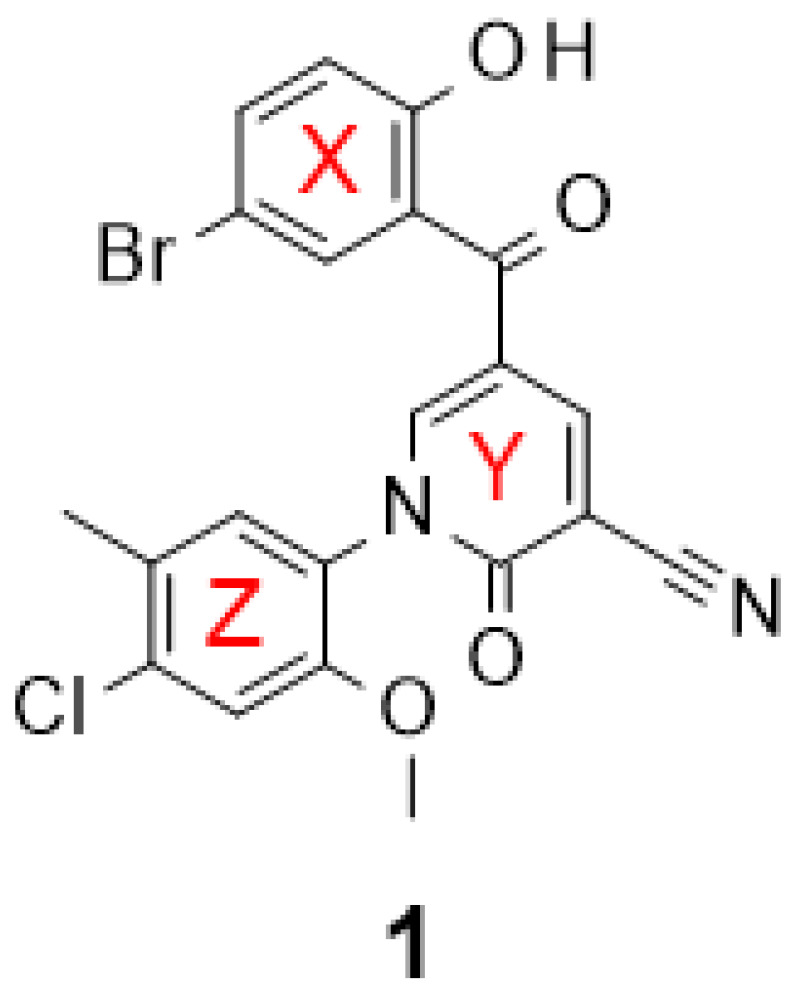
Structure of 5-(5-bromo-2-hydroxybenzoyl)-1-(4-chloro-2-methoxy-5-methylphenyl)-2-oxo-1,2-dihydropyridine-3-carbonitrile, an inhibitor of the human Caf1/CNOT7 nuclease. The reported IC_50_ value is 14.6 ± 3.1 μM [37].

Previously, we carried out a virtual screen (83,086 compounds) to identify inhibitors of the Caf1/CNOT7 nuclease and assayed the top 1440 ranked compounds using a fluorescence-based assay [37]. Here, we disclose the structure of the most potent compound of the screen, 5-(5-bromo-2-hydroxybenzoyl)-1-(4-chloro-2-methoxy-5-methylphenyl)-2-oxo-1,2-dihydropyridine-3-carbonitrile (**1**, Figure 1). The activity of the compound was confirmed in a gel-based assay and shown to be selective to Caf1/CNOT7 [37].

To confirm the activity of **1**, we re-purchased the compound and confirmed inhibition of the Caf1/CNOT7 enzyme. The obtained IC_50_ (24.1 ± 3.3 µM, *n* = 3) conformed with the originally determined value (14.6 ± 3.1 µM, *n* = 3) (*p* = 0.104, two-tailed, unpaired *t*-test) (Table 1).

**Figure 2:**
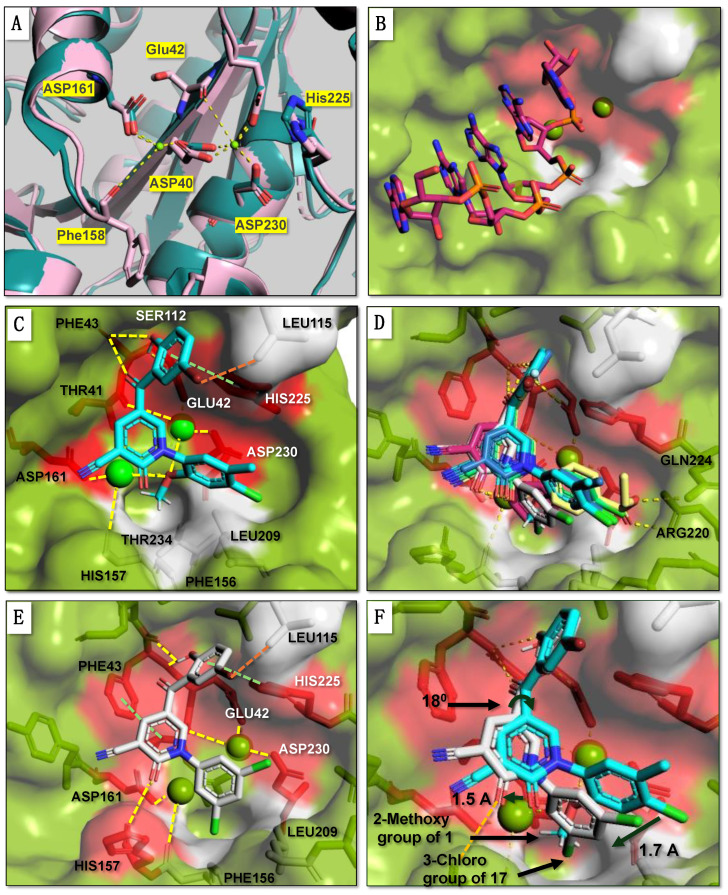
Molecular docking of inhibitors into the active site of human Caf1/CNOT7. (**A**) Catalytic site of the Caf1/CNOT7 enzyme. Shown is the position of the residues coordinating two Mg^2+^ ions (bright green) in the active site of *Schizosaccharomyces pombe* Pop2 protein (PDB 2P51, slate blue) [9] and the corresponding coordinating residues of human Caf1/CNOT7 (PDB 7VOI, salmon red) [12]. (**B**) Model of human Caf1/CNOT7 bound to poly(A) RNA. The RNA was obtained by superposition of the structure of human Caf1/CNOT7 (PDB 7VOI) [12] and *Schizosaccharomyces pombe* Pan2 in complex with poly(A) RNA (PDB 6R9J) [45]. Shown are the surface views of the residues developing; polar interactions (red) and nonpolar interactions (white) with the analogues (**1**, **8**, **9**, **11**, **15** and **17**) in the active site. (**C**) Molecular docking of **1** (cyan) into the active site of Caf1/CNOT7. (**D**) Overlay of **1** (cyan) with the plausible conformation of the five most potent analogues **8** (light green), **9** (light yellow), **11** (slate blue), **15** (magenta) and **17** (white). (**E**) Molecular docking of **17** (white) into the active site of Caf1/CNOT7 (**F**) Overlay of **1** (cyan) with the plausible conformation of the most potent analogue **17**, into the active site of Caf1/CNOT7.

To obtain insight into the probable binding mode of **1**, we used molecular docking using the X-ray structure of human Caf1/CNOT7 in complex with the MIF4G domain of CNOT1 and Ccr4/CNOT6L (PDB 7VOI) [12]. Because the active site Mg^2+^ ions were not resolved in this structure, the metal ions were transposed from the structure of the homologous *Schizosaccharomyces pombe* Pop2 protein, which displays a highly similar conformation of the coordinating side chains of amino acids corresponding to Asp40, Glu42, Asp161 and Asp230 (Figure 2A). Next, the position of the RNA substrate was modelled by superimposing the structures of Caf1/CNOT7 and the DEDDh-type *Schizosaccharomyces pombe* Pan2 ribonuclease in complex with oligo(A) RNA (PDB 6R9J) [45] (Figure 2B, Supplementary Figure S2A). To assess the effectiveness of the docking approach, we initially redocked a native ligand, poly(A) RNA, into the active site of human Caf1/CNOT7. To this end, the ligand from the crystal structure was retrieved and redocked resulting in a binding orientation closely matching the original pose (docking score, −8.4 kcal/mol, RMSD 1.6Å) (Supplementary Figure S2B).

Molecular docking of compound **1** indicated that its probable position in the active site blocks access of the substrate (Figure 2C). Plausible interactions between **1** and the Caf1/CNOT7 protein primarily include polar contacts between the benzoyl group and the residues Glu42, Phe43, His225, Asp161 and Asp230 (Figure 2C, red surface) and nonpolar contacts between the methoxy group and residues Phe156, Leu209 and Thr234 (Figure 2C, grey surface). Here, the backbone amide of Phe43 forms hydrogen bonds with the 2-hydroxyl and 1-carbonyl groups of the benzoyl moiety of **1**. The aromatic phenyl (Figure 1, ring X) of the benzoyl moiety develops Van der Waals interactions with the residues Ser112, Leu115 and His225, in addition to Van der Waals interactions between the 5-bromo group and Ser112. His225 also forms T-shaped π-π interactions with both the benzoyl and the 1-phenyl ring structures of **1**. In the case of the 2-methoxy substitution on the phenyl (Figure 1, ring Z), the methyl group protrudes to the lipophilic trough between the residues Phe156, Leu209 and Thr234. Moreover, the methoxy oxygen coordinates interactions with two Mg^2+^ ions that mutually coordinate cohesive ionic bonds with other protein residues, including Thr41-Mg^2+^(OMe)-Asp230 and Asp161-Mg^2+^(OMe)-His157.

Based on the probable interactions between **1** and the Caf1/CNOT7 nuclease, we hypothesised that changing the position of substituents on the 1-phenyl group or the introduction of alternative substituents may lead to additional Van der Waals interactions. We therefore acquired 15 analogues of **1** with different substituents on the 1-phenyl group (Figure 1, ring Z) and determined their Caf1/CNOT7 nuclease inhibitory potential (IC_50_ values) to establish a preliminary structure–activity relationship (Table 1). All compounds were selected based on their predicted drug-likeness, favourable predicted pharmacokinetic profile, and accessibility to synthetic chemistry (Supplementary Tables S2–S4). The activity of **8** and **15** indicates that the 4-chloro and 3-methyl substituents appear to contribute to binding. The 2-methoxy group alone does not appear to contribute significantly as evident from the reduced potency of **6**. Moderate differences in potency were observed for the remaining compounds with one or two substituents. Only compound **17**, which contains 3, 5-dichloro substituents on the 1-phenyl group, displayed a lower IC_50_ value than **1**, although the difference was not statistically significant (*p* = 0.9914). Compound **12**, which has no substituents on the 1-phenyl moiety, displayed a 4–5-fold lower potency compared to **1** (*p* < 0.0001), indicating that substituents make a significant contribution to potency.

In addition to compounds with different substituents in the 1-phenyl group, we also acquired an analogue of **1** containing a 5-fluoro atom instead of a 5-bromo substituent on the benzoyl moiety (**11**). Compound **11** displayed a modest (<2-fold) reduction in potency compared to **1**, which was not statistically significant (*p* = 0.517). Perhaps the larger atom size of the 5-bromo substituent compared to the 5-fluoro substituent may increase the probability of Van der Waals interactions with adjoining protein residues.

To understand the activity of the analogues, we also evaluated the probable binding mode of the five most potent analogues **8**, **9**, **11**, **15**, and **17** using molecular docking (Figure 2D, Supplementary Table S1). In all cases, the probable conformation of the benzoyl group adopts a similar conformation as observed for **1** (Figure 2D). However, some differences in the position of the central pyridone ring (Figure 1, ring Y) and the 1-phenyl group (Figure 1, ring Z) were observed. The probable interactions and conformations of **8** and **11** were very similar to that of **1**. In case of **9**, however, the oxygen and carbonyl of the 4-ethoxycarbonyl group forms possible hydrogen bonds with the side chains of Arg220 and Gln224, respectively.

More significant differences were observed for the probable interactions of **15** and **17** with active site residues. In these cases, His225 makes T-shaped π-π interactions with the benzoyl ring while Phe43 forms π-π interactions with the central pyridone ring (Figure 1, ring Y) in addition to a probable hydrogen bond between the Phe43 backbone amide-NH and the 2-hydroxyl and 1-carbonyl groups of the benzoyl group. The benzoyl ring (Figure 1, ring X) forms Van der Waals interactions with the residues Phe43, Ser112, Leu115 and His225, and in case of **17**, also with Phe156 (Figure 2E).

When compared to **1** (Figure 2E,F), the central pyridone ring (Figure 1, ring Y) of **17** may swing clockwise by 18° thereby shifting the 3,5-dichlorophenyl ring (Figure 1, ring Z) by 1.7 Å so that the 3-chloro group takes the position of 2-methoxy group of **1**. This orientation also moves 2-ketone group of the pyridone ring (Figure 1, ring Y) of **17** by 1.5 Å towards His157 to form a polar interaction with its carbamide oxygen. The docking scores of the selected analogues showed a moderate correlation with experimental activities (IC_50_) (Supplementary Figure S2C).

In conclusion, we disclose that 5-(5-bromo-2-hydroxy-benzoyl)-1-(4-chloro-2-methoxy-5-methyl-phenyl)-2-oxo-pyridine-3-carbonitrile is an inhibitor of the Caf1/CNOT7 nuclease. The activity of 16 structural analogues was determined. Substituents on the 1-phenyl moiety contributed to the interaction with the Caf1/CNOT7, but no compound was identified with a statistically significant increase in inhibitory activity towards the Caf1/CNOT7 nuclease.

## 3. Materials and Methods

Compounds (purity > 95%) were obtained from Enamine. Purity of the compounds was verified by LC-MS. The inhibitory activity of the compounds was assessed using a fluorescence-based activity assay described before [37,46]. Briefly, purified Caf1/CNOT7 was pre-incubated in the presence of compound or vehicle only (DMSO) before addition of a Fluorescein-labelled RNA oligonucleotide substrate. The composition of the final reaction mixture (10 µL) was: 0.4 µM Caf1/CNOT7, 1 µM Flc-RNA substrate, 20 mM Tris–HCl pH 7.9, 50 mM NaCl, 2 mM MgCl_2_, 10% glycerol, 1 mM β-mercaptoethanol and 5% DMSO. After incubation at 30 °C for 60 min, 10 µL probe mix (5 µM TAMRA-DNA probe, 1% SDS and 20 mM Tris–HCl pH 8.0, 0.5 mM EDTA) was added. Fluorescence intensity was measured using a BioTek Synergy HT plate reader (Agilent, Cheshire, UK) using the filters 485 ± 20 nm (excitation) and 528 ± 20 nm (emission). Raw data were analysed in Microsoft Excel and non-linear regression was carried out using GraphPad Prism 6. Statistical analysis (one-way ANOVA with post-hoc Dunnett’s test) was also carried out using GraphPad Prism.

### Molecular Docking

Molecular docking was carried out using Autodock Vina version 1.2 and UCSF Chimera [43,44,47]. The human Caf1/CNOT7 protein for docking was prepared from the X-ray structure of Caf1/CNOT7 in complex with CNOT1 and Ccr4/CNOT6L (PDB 7VOI, chain B) [12] by removal of ions and water molecules. Subsequently, the positions of Mg^2+^ ions were transposed from the active site of the *Schizosaccharomyces pombe* orthologue Pop2 (PDB 2P51) [9]. The preparation of PDBQT files for the protein was done via UCSF Chimera [47], which was used to assign polar hydrogens and Kollman charges. The grid size was set to 25 × 25 × 25 Å^3^ centred around the active site Mg^2+^ ion coordinated by Asp40, Glu42 and Asp230 (coordinates 136, 10, 270). Ligands were built in UCSF Chimera. Following energy minimisation, partial charges were calculated using the Gasteiger method. After docking, poses with a root mean square difference (RMSD) of less than 1.0 Å were clustered and represented by the pose with the most favourable free energy of binding. Ligand poses were analysed using UCSF Chimera and the PoseEdit algorithm accessed through the Proteins Plus portal [48,49]. The receptor-ligand poses were visualised using PyMol [50].

## Figures and Tables

**Table 1:** The inhibitory activity of 5-benzoyl-2-oxo-1-phenyl-1,2-dihydropyridine-3-carbonitrile analogues versus Caf1/CNOT7 ^a^.

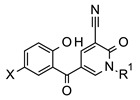				Docking Score ^b^	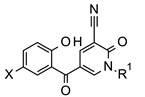				Docking Score ^b^
Cmpd	X	R^1^	IC_50_ (μM) ^a^		Cmpd	X	R^1^	IC_50_ (μM) ^a^	
**1**	Br	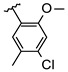	24.1 ± 3.3 (*n* = 3) ^c^	−8.17					
**2**	Br	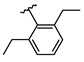	39.4 ± 3.1 (*n* = 4)	-	**10**	Br	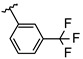	42.1 ± 1.4 (*n* = 4)	-
**3**	Br	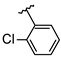	65.6 ± 3.3 (*n* = 4)	-	**11**	F	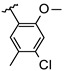	33.6 ± 1.1 (*n* = 4)	−8.40
**4**	Br	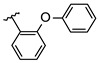	39.3 ± 1.6 (*n* = 4)	-	**12**	Br	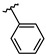	108.3 ± 4.0 (*n* = 4)	−8.11
**5**	Br	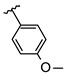	81.1 ± 5.0 (*n* = 4)	-	**13**	Br	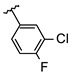	47.0 ± 2.7 (*n* = 4)	-
**6**	Br	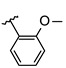	80.9 ± 9.2 (*n* = 4)	-	**14**	Br	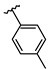	57.5 ± 4.7 (*n* = 4)	-
**7**	Br	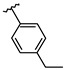	39.0 ± 2.6 (*n* = 4)	-	**15**	Br	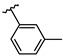	31.8 ± 1.8 (*n* = 3)	−9.06
**8**	Br	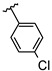	32.2 ± 1.6 (*n* = 4)	−8.53	**16**	Br	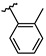	44.5 ± 3.6 (*n* = 3)	-
**9**	Br	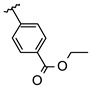	33.2 ± 0.5 (*n* = 4)	−8.97	**17**	Br	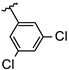	19.2 ± 1.1 (*n* = 3)	−8.72

^a^ Activity was determined versus the human nuclease enzyme Caf1/CNOT7 as described [37]. IC_50_s are the mean ± standard error. ^b^ The docking score (kcal/mol) was calculated using Autodock Vina [43,44]. ^c^ The originally reported IC_50_ value is: 14.6 ± 3.1 μM (*n* = 3) [37].

## Data Availability

Data are contained within the article and Supplementary Materials.

## Supplementary Materials

The following supporting information can be downloaded at: https://www.mdpi.com/1420-3049/29/18/4351/s1. References [9,12,45,48,51,52,53] are citied in the Supplementary Materials.
