# Supplementary material for: Discovery of Substituted 5-(2-Hydroxybenzoyl)-2-Pyridone Analogues as Inhibitors of the Human Caf1/CNOT7 Ribonuclease
Source: Molecules. 2024 Sep 13;29(18):4351. doi: 10.3390/molecules29184351 (PMC11870035; doi:10.3390/molecules29184351)
Supplement: Supplementary file 1 [file molecules-29-04351-s001.zip › molecules-3050773-supplementary.pdf]

Supplementary Data

The Discovery of Substituted 5-(2-Hydroxybenzoyl)-2-Pyridone Analogues as Inhibitors of the Human Caf1/CNOT7 Ribonuclease

Ishwinder Kaur, Gopal P. Jadhav *,†, Peter M. Fischer * and Gerlof Sebastiaan Winkler *

School of Pharmacy and Centre for Biomolecular Sciences, University of Nottingham, Nottingham NG7 2RD, UK; ishwinder.kaur@ntu.ac.uk

* Correspondence: gopaljadhav@creighton.edu (G.P.J.); paapf1@exmail.nottingham.ac.uk (P.M.F.); sebastiaan.winkler@nottingham.ac.uk (G.S.W.)

† Current address: School of Medicine, Creighton University, Omaha, NE 68178, USA.

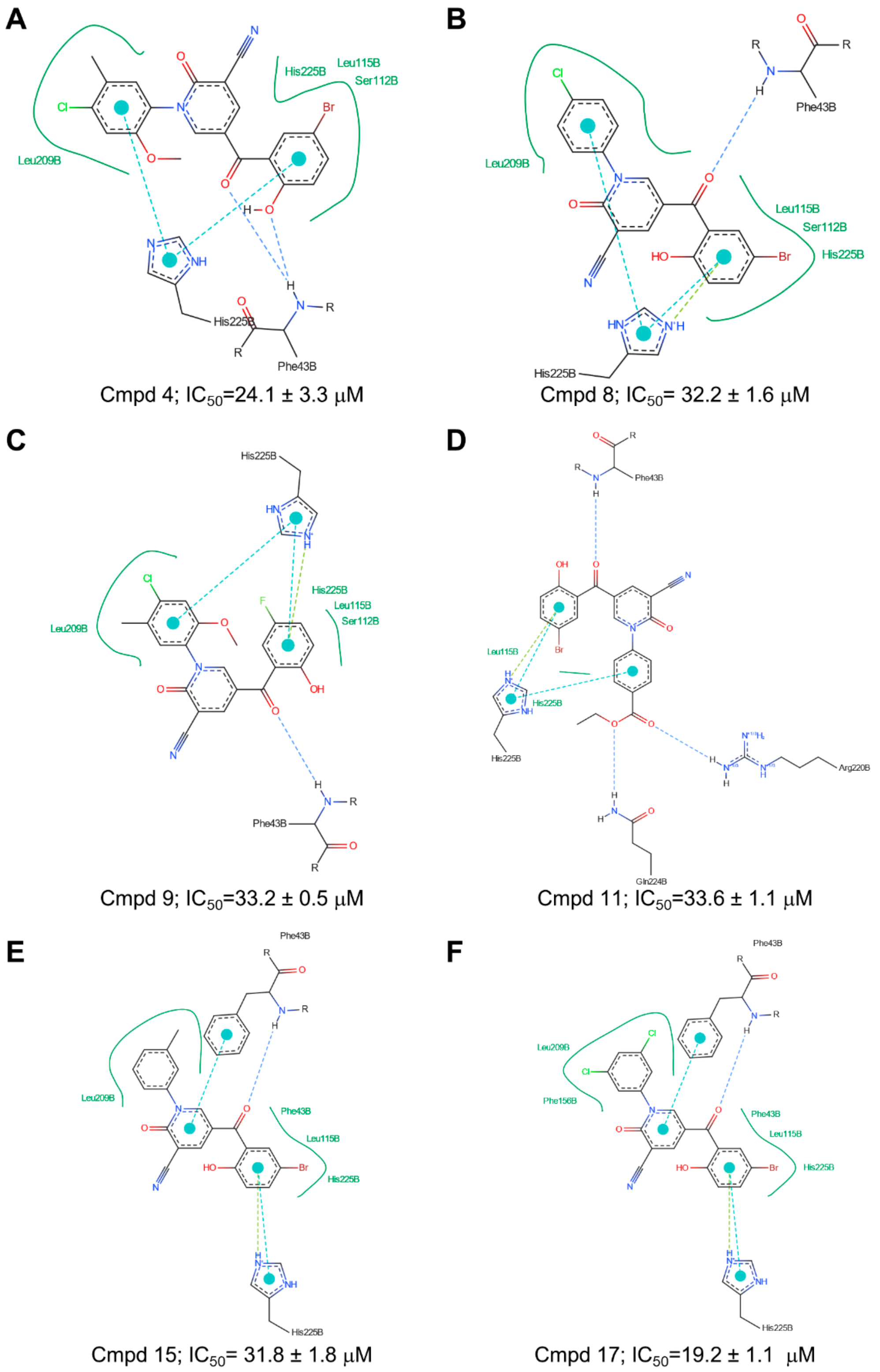

Supplementary Figure S1. Analysis of main inhibitor - Caf1/CNOT7 interactions. (A-F) Docking poses were analysed using the PoseEdit algorithm [45], which generated the 2D schematics. PoseEdit was accessed via the Proteins Plus portal (https://proteins.plus).

Supplementary Table S1. Summary of amino acids of Caf1/CNOT7 involved in main interactions.[a]

Cmpd	IC_{50} (μM) (mean±sem)	Hydrogen bonds	π-π interactions	Van der Waals contacts
**4**	24.1 ± 3.3	Phe43	His225	His225<br>Leu209<br>Leu115<br>Ser112
**8**	32.2 ± 1.6	Phe43	His225	His225<br>Leu209<br>Leu115<br>Ser112
**9**	33.2 ± 0.5	Gln224<br>Arg220<br>Phe43	His225	His225<br>Leu115
**11**	33.6 ± 1.1	Phe43	His225	His225<br>Leu209<br>Leu115<br>Ser112
**15**	31.8 ± 1.8	Phe43	His225<br>Phe43	His225<br>Leu209<br>Leu115<br>Phe43
**17**	19.2 ± 1.1	Phe43	His225<br>Phe43	His225<br>Leu209<br>Phe156<br>Leu115<br>Phe43

[a] Docking positions were analysed using the PoseEdit package accessed through the Proteins Plus portal (https://proteins.plus) [45]. Only amino acids involved in main interactions are listed.

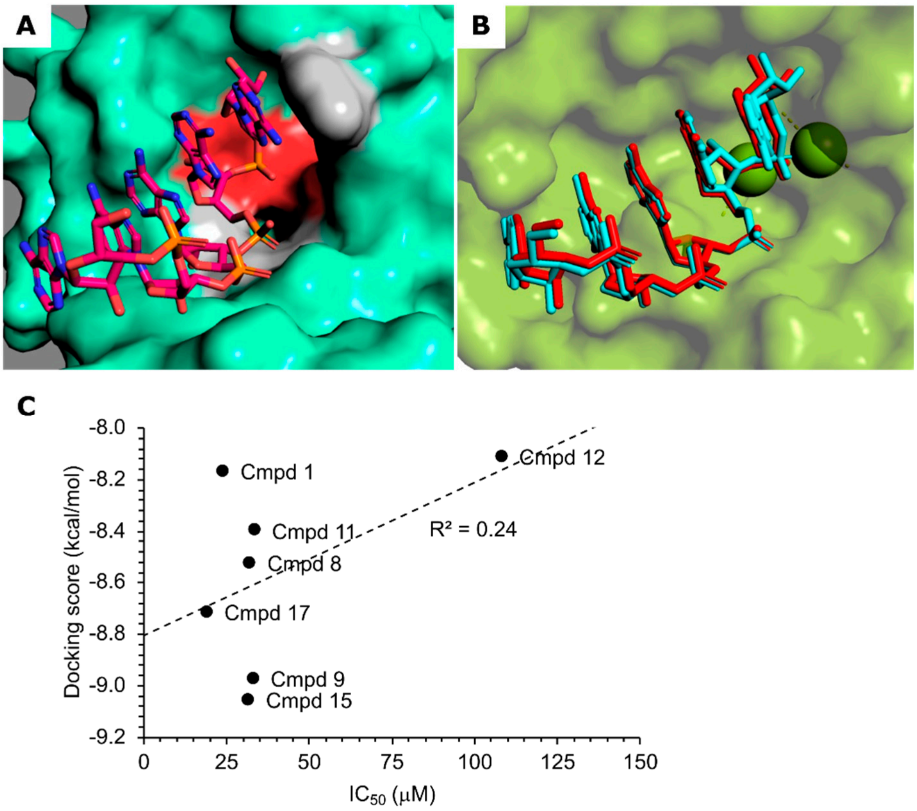

Supplementary Figure S2. Docking validation. (A) Poly(A) RNA (salmon red, stick view) in complex with *Schizosaccharomyces pombe* Pan2 (PDB 6R9J; cyan, surface view) [45]. **(B)** Overlay of re-docked poly(A) RNA (cyan stick view) and poly(A) RNA (salmon red stick view) superimposed onto catalytic site of human Caf1/CNOT7 (PDB 7VOI; green space view) [12]. The active site Mg^{2+} (superimposed from PDB 2P51 [9]) are indicated as green spheres. **(C)** Moderate correlation between experimental IC_{50} and docking scores. Linear regression is shown as dotted line, while the correlation coefficient (R^2) shown.

Supplementary Table S2. Physicochemical properties [51-53]

Cmpd	Formula	MW	#Heavy atoms	#Rotatable bonds	#H-bond acceptors	#H-bond donors	MR	TPSA	XLOGP3	MLOGP	ESOL Log S	ESOL Solubility (mg/ml)	ESOL Class
1	C21H14BrClN2O4	473.7	29	4	5	1	112.82	92.32	4.79	2.56	-5.99	4.85E-04	Moderately soluble
2	C23H19BrN2O3	451.31	29	5	4	1	115.9	83.09	5.42	3.04	-6.18	2.97E-04	Poorly soluble
3	C19H10BrClN2O3	429.65	26	3	4	1	101.36	83.09	4.45	2.67	-5.62	1.03E-03	Moderately soluble
4	C25H15BrN2O4	487.3	32	5	5	1	122.87	92.32	5.35	2.97	-6.46	1.70E-04	Poorly soluble
5	C20H13BrN2O4	425.23	27	4	5	1	102.84	92.32	3.8	1.86	-5.1	3.38E-03	Moderately soluble
6	C20H13BrN2O4	425.23	27	4	5	1	102.84	92.32	3.8	1.86	-5.1	3.38E-03	Moderately soluble
7	C21H15BrN2O3	423.26	27	4	4	1	106.12	83.09	4.62	2.62	-5.6	1.05E-03	Moderately soluble
8	C19H10BrClN2O3	429.65	26	3	4	1	101.36	83.09	4.45	2.67	-5.62	1.03E-03	Moderately soluble
9	C22H15BrN2O5	467.27	30	6	6	1	112.44	109.39	4.04	2.23	-5.33	2.18E-03	Moderately soluble
10	C20H10BrF3N2O3	463.2	29	4	7	1	101.35	83.09	4.71	3	-5.87	6.18E-04	Moderately soluble
11	C21H14ClFN2O4	412.8	29	4	6	1	105.08	92.32	4.2	2.34	-5.24	2.37E-03	Moderately soluble
12	C19H11BrN2O3	395.21	25	3	4	1	96.35	83.09	3.82	2.18	-5.03	3.67E-03	Moderately soluble
13	C19H9BrClFN2O3	447.64	27	3	5	1	101.32	83.09	4.55	3.05	-5.78	7.48E-04	Moderately soluble
14	C20H13BrN2O3	409.23	26	3	4	1	101.32	83.09	4.19	2.4	-5.33	1.91E-03	Moderately soluble
15	C20H13BrN2O3	409.23	26	3	4	1	101.32	83.09	4.19	2.4	-5.33	1.91E-03	Moderately soluble
16	C20H13BrN2O3	409.23	26	3	4	1	101.32	83.09	4.19	2.4	-5.33	1.91E-03	Moderately soluble
17	C19H9BrCl2N2O3	464.1	27	3	4	1	106.37	83.09	5.08	3.16	-6.21	2.84E-04	Poorly soluble

Supplementary Table S3. ADME properties [51-53]

Compd	GI absorption	BBB permeant	Pgp substrate	CYP1A2 inhibitor	CYP2C19 inhibitor	CYP2C9 inhibitor	CYP2D6 inhibitor	CYP3A4 inhibitor	log Kp (cm/s)	Lipinski #violations	Bioavailability Score
**1**	High	No	No	No	Yes	Yes	No	Yes	-5.79	0	0.55
**2**	High	No	No	No	Yes	Yes	No	Yes	-5.2	0	0.55
**3**	High	No	No	No	Yes	Yes	No	No	-5.76	0	0.55
**4**	High	No	No	No	Yes	Yes	No	No	-5.47	0	0.55
**5**	High	No	No	No	No	Yes	No	Yes	-6.2	0	0.55
**6**	High	No	No	No	No	Yes	No	Yes	-6.2	0	0.55
**7**	High	No	No	Yes	Yes	Yes	No	No	-5.6	0	0.55
**8**	High	No	No	Yes	Yes	Yes	No	No	-5.76	0	0.55
**9**	High	No	No	No	Yes	Yes	No	Yes	-6.28	0	0.55
**10**	High	No	No	No	Yes	Yes	No	No	-5.78	0	0.55
**11**	High	No	No	No	No	Yes	No	Yes	-5.84	0	0.55
**12**	High	No	No	Yes	No	Yes	No	No	-6	0	0.55
**13**	High	No	No	No	Yes	Yes	No	No	-5.8	0	0.55
**14**	High	No	No	Yes	No	Yes	No	No	-5.82	0	0.55
**15**	High	No	No	Yes	No	Yes	No	No	-5.82	0	0.55
**16**	High	No	No	Yes	No	Yes	No	No	-5.82	0	0.55
**17**	High	No	No	No	Yes	Yes	No	No	-5.52	0	0.55

Supplementary Table S4. Medicinal chemistry accessibility [51-53]

Compd	PAINS #alerts	Brenk #alerts	Leadlikeness #violations	Synthetic Accessibility
**1**	0	0	2	3.03
**2**	0	0	2	3.12
**3**	0	0	2	2.71
**4**	0	0	2	3.25
**5**	0	0	2	2.81
**6**	0	0	2	2.89
**7**	0	0	2	2.9
**8**	0	0	2	2.69
**9**	0	0	2	3.03
**10**	0	0	2	2.84
**11**	0	0	2	3.01
**12**	0	0	2	2.68
**13**	0	0	2	2.71
**14**	0	0	2	2.79
**15**	0	0	2	2.79
**16**	0	0	2	2.77
**17**	0	0	2	2.73

References

9. Jonstrup AT, Andersen KR, Van LB, Brodersen DE (2007) The 1.4-Å crystal structure of the *S. pombe* Pop2p deadenylase subunit unveils the configuration of an active enzyme. *Nucleic Acids Res* **35**:3153-3164. doi: 10.1093/nar/gkm178

12. Zhang Q, Pavanello L, Potapov A, Bartlam M, Winkler GS (2022) Structure of the human Ccr4-Not nuclease module using X-ray crys-tallography and electron paramagnetic resonance spectroscopy distance measurements. *Protein Sci* **31**:758-764. doi: 10.1002/pro.4262

45. Tang TTL, Stowell JAW, Hill CH, Passmore LA (2019) The intrinsic structure of poly(A) RNA determines the specificity of Pan2 and Caf1 deadenylases. *Nat Struct Mol Biol* **26**:433-442. doi: 10.1038/s41594-019-0227-9

48. Diedrich K, Krause B, Berg O, Rarey M (2023) PoseEdit: enhanced ligand binding mode communication by interactive 2D diagrams. *J Comput Aided Mol Des* **37**:491-503. doi: 10.1007/s10822-023-00522-4.

51. SwissADME: a free web tool to evaluate pharmacokinetics, drug-likeness and medicinal chemistry friendliness of small molecules. Sci. Rep. (2017) 7:42717.

51. iLOGP: a simple, robust, and efficient description of n-octanol/water partition coefficient for drug design using the GB/SA approach. J. Chem. Inf. Model. (2014) 54(12):3284-3301.

53. A BOILED-Egg to predict gastrointestinal absorption and brain penetration of small molecules. ChemMedChem (2016) 11(11):1117-1121.
